# Dancing with Disease: A Dancer’s Reflections on Moving with People with Parkinson’s and Memory Loss

**DOI:** 10.3389/fneur.2016.00137

**Published:** 2016-08-25

**Authors:** David William Marchant

**Affiliations:** ^1^Performing Arts Department, Washington University in St. Louis, St. Louis, MO, USA

**Keywords:** dance, dance therapy, therapy, Parkinson’s disease, Alzheimer’s disease, falling, improvisation, exercise

## Introduction: Field Notes from an Experiential Movement Researcher

I am a professional dancer, choreographer, and Alexander Technique instructor. I joined the Performing Arts faculty at Washington University in St. Louis in 1994, where I teach contemporary concert art dance and somatic practices. Dancing is fundamentally a study of coordination, balance, and movement control. Because these essential goals are shared in movement therapies, I have become interested in contributing knowledge gained by dancing and collaboration with scientists working on therapeutic interventions. I am particularly interested in evidence that, for people attempting to mitigate symptoms of Parkinson’s disease (PD), dancing in a variety of forms is emerging in scientific literature as an effective approach (1, 2). Furthermore, dancing is valuable to people who do it, not only for cognitive and motor issues but for social and personal fulfillment, while living with PD (3). I am also interested in correlative evidence suggesting that dancing, as a lifestyle activity, may confer a protective effect against risk of dementia (4). In this article, I would like to share several personal insights regarding dancing and why I believe it is so effective.

In 2008, I met Madeleine Hackney and Gammon Earhart who were engaged in a series of studies demonstrating that Argentine Tango improved balance and functional mobility in people with PD (5–7). I was invited to create and administer an untested intervention for PD using “contact improvisation” (8). We showed similar improvements as the Tango pilot study. However, participants reported preferring improvising (not having to learn step patterns), and the increased human touch that the practice fostered became valuable to them. This report in the participant survey is among the most meaningful to me; I firmly believe that live human touch is an essential ingredient in physical, mental, and emotional health.

During this past year, my students and I conducted a dozen non-scientific workshops for people with Alzheimer’s disease (AD), applying creative dance practices I learned from dance artist Liz Lerman (9).

Memories are not just “in our heads,” they are whole-person, embodied experiences – what I call “corps memories.” I am intrigued by evidence that integrating a motoric component such as pantomimic gesture or sign language during learning improves memory retention (10, 11). In workshops, people shared memories, and we created dance from the spontaneous gestural movements that typically accompany speech when they become immersed in reverie. This organic combination of narrative and kinesthetic sensation seems to enhance the details of memory. Artistically, there is something poignant, “dancing” memories that will be lost.

## Looking Backwards, with Ideas for Moving Forwards

When I began these workshops, I never knew what to expect. Perhaps, it was a virtue, I had so few assumptions about what people with PD and AD “can’t” do. We simply started where we were and pushed the edge of our ability gradually, improvising and adapting along the way. Participants did movements that I would not have thought older adults would do, much less with disease. In the process, I think we all surprised ourselves, and that felt empowering to me and I believe to them as well.

Dancing with elders has changed the way I understand movement. Studying movement by feeling it from the inside has generated numerous ideas and questions. From this point of view, I will discuss several themes for scientists to consider with possibilities for further investigation: (1) how subjective, experiential research can contribute knowledge about movement complementary to objective science; (2) how improvised movement activity fosters adaptive capacity useful for recovering falls during unexpected loss of balance; (3) that dancing is more complex than “exercise,” engaging cognitive, neuromuscular, esthetic, and social aspects of a person, integrally. Finally, I propose more collaboration between disciplines with a broad lens toward the goals of therapy, including how dancing offers meaningful ways to live with disease.

## Objective and Subjective Evidence are Complementary

“More evidence is needed;” I like this understatement deployed by scientists to estimate our insufficient understanding (and to nudge for continued funding). I suggest that more *kinds* of evidence are needed to fully explore human functioning. Objective measurement does not always capture the meaning of the lived experience. Subjective, experiential study can add different information and help point our investigations in new directions. Dancing is highly complex. It will be difficult to ever fully analyze why it is so widely beneficial, but the evidence that we feel good doing it is well documented.

Subjective experience is not as highly trusted as objective evidence. I understand why we feel less confident of data “corrupted” by non-quantifiable things like feelings. I argue subjectivity is not inherently unreliable; we only need more advanced education of sensation to train skills for reliable subjective self-assessment. Somatic and artistic practices offer such training and experiential methods of critical thinking and research in human movement (12).

Furthermore, subjectivity is where we live, and the way we feel is often what counts most to a person. For people with PD and AD, in my workshops, evidence that dance is beneficial is encouraging, but I find they mainly do it because they feel better and enjoy moving together. That is evidence enough it is worth doing.

## Improvisation is an “Active Ingredient”

Studying movement from inside the sensory experience, I find that improvising develops superior motor control. In two studies of dance interventions for PD, I see a hint that improvising may be an “active ingredient” in their effectiveness (8, 13). Like real life, improvised movement generates dynamic, unpredictable interactions with the environment and/or people; this fosters capacity for adaptive response critical to successful living. Even rote activities feel “new,” requiring adaptive response during the learning phase. But, in time, this virtue may fade, as the activity requires less attention to repeat. I am curious to see further investigation of “interactivities” that remain variable and challenge people to cope with the unpredictability of living, rather than avoid such risks with routines.

I also wish to correlate improvisation with Ellen Langer’s research reporting wide ranging cognitive, emotional, and physical benefits of being “mindful” in novel ways (14–16). Her instructions to experimental, “mindful” groups often take the form of “thought experiments,” teaching them to attend and improvise tasks in new ways (17). Improvising induces, if not requires, the quality of presence that Langer is recommending, suggesting that improvised interventions may be more beneficial than routine ones.

## Falling is Not an Error, it is a Skill

One test of adaptive capacity appears when a person falls. Falling un-injuriously requires improvised adaptation to correct unexpected loss of balance. When I asked study participants, in 2010, about the risk of falling, they reported that their physicians said, “don’t fall.” Evidence shows that fear of falling may actually increase fall occurrence (18). Based on personal experience, trying not to fall is a terrible strategy for balancing, causing high muscle tension and overcorrection errors that ironically lead to falling. I find the secret to balance is becoming at ease with falling, fluidly oscillating between falling and recovering (a principle I learned in techniques by mid-century modern dance artist Doris Humphrey).

In Marchant et al. (8), we reported, “All together, strategies were intended to teach participants that stability in balance is a skill of continuous adaptive movement, rather than of fixity, holding or prevention of movement. Rather than suggesting participants try to ‘avoid falling,’ this workshop taught them how to fall more safely” (8). This is also a fundamental principle and technique in contact improvisation dancing.

Since publishing that statement, I have come to the opinion that falling is not an “error” we should avoid – it is *the* skill we should develop and maintain. Like Humphrey, my definition of “falling” includes weight transfers involved in walking; in this model, safely “losing” balance becomes equally fundamental to movement. I find that dancers’ balance (suspension) becomes calmer and more natural when they are taught to allow their weight to “fall outward, in all directions equally.” Corrections are achieved by falling in the opposite direction of an imbalance. When locomotion is desired, releasing counterbalance allows the body to move in the intended direction. In this approach, both suspension and motion are achieved with the same strategy, easing muscle tension instead of tightening, producing smoother transfers with fewer overcorrection errors.

Participants also said that it was not a matter of *if* they fall, but *when*. If the default prescription is “don’t fall,” there may be insufficient consideration or training of *how* a person should respond when they do fall. In our intervention, people were challenged to shift weight off center intentionally. The more familiar they became with instability, the more confidently they improvised fluid recoveries to perturbations.

I suggest we look closely at how we define and measure balance with care to not unintentionally send a message that good balance is something held still or that our balance is worse if we are moving to adjust.

## Why Dance is so Beneficial: More than Mere “Exercise”

When we think of exercise as primarily muscle strengthening, cardiovascular conditioning, or even neuromuscular activity, we potentially miss something whole about the experience of moving. I suspect the complexity of dynamically interactive dependent variables that dancing introduces is not only why it is difficult to tease out causal relationships but also why it is so effective. It is tricky trying to isolate variables for “scienceability” without denaturing activities to the point that they lose their inherent value.

Dancing works on the whole person. Complex esthetic movement fosters relationships between brain areas, stimulating “mindbody” to work in novel, “more-than-necessary,” neurogenerative ways. I also believe that esthetics – intangibles like “beauty” and “how it feels” – are not merely superficial embellishments. Appealing sensory experience comingled with conscious attention in movement integrates brain functioning. Exercise can become rote, reductive, and repetitive, performed in ways that diminish esthetic appeal, and, with it, the need/desire for cognitive engagement. I see people walking on treadmills listening to their iPod, reading a book on the easel, with a TV on in the background. If we need three distractions from how bored we feel, benefits of such exercise may be analogous to taking a multivitamin, but no substitute for healthy eating. I argue that cognitive presence is an essential nutrient of “whole” movement.

## More Collaboration Between Disciplines is Needed

What I know about human movement I know by moving with people in my classroom “laboratory.” Collaborating with dancers, I have learned ways to improve coordination, balance, and motor control. Collaborating with scientists, I learned that my knowledge about human movement might have valuable applications. And with scientific methods, I learned I could rationally seek more evidence to support my subjective hunches.

I predict that dancing will continue to emerge as a preferred therapeutic intervention and preventative activity for people with a wide variety of illness conditions. In support of this trend, I recommend scientists seek more collaboration with dancers; they may not yet fully realize that what they know is of value to you. Dancers interested in movement therapy may consider studying movement science. And I encourage scientists to dance. Dancing is not just something one watches, it is an experience. The sensation of moving cannot be replaced with objective observation, and if scientists are having these experiences, I am confident that they will generate new ideas for investigation.

## People with Disease are Not Just Degenerating, They are Alive

Moving with elders has changed my definition of “dance” and what is “beautiful.” The people I have worked with are some of the most alive people I have ever met – fully engaged and present. Brené Brown writes, “It didn’t take long for me to learn that … for many of us, there is no form of self-expression that makes us feel more vulnerable than dancing. Its literally full-bodied vulnerability” (19). But in my experience, whatever vulnerability people with PD feel about dancing pales compared with the decline they are already facing. They are not as nearly as self-conscious or inhibited as many of my college students. Witnessing a person moving at the edge of their ability, whatever that may be, is what is most beautiful to me.

## Author Contributions

DM is the sole author of this opinion article and has contributed all of its content.

## Conflict of Interest Statement

The author declares that the research was conducted in the absence of any commercial or financial relationships that could be construed as a potential conflict of interest.
